# GATmath and GATLc: Comprehensive benchmarks for evaluating Arabic large language models

**DOI:** 10.1371/journal.pone.0329129

**Published:** 2025-09-02

**Authors:** Safa AlBallaa, Nora AlTwairesh, Abdulmalik AlSalman, Sultan Alfarhood

**Affiliations:** 1 Department of Computer Science, College of Computer and Information Sciences, King Saud University, Riyadh, Saudi Arabia; 2 Research Chair of Online Dialogue and Cultural Communication, Department of Computer Science, College of Computer and Information Sciences, King Saud University, Riyadh, Saudi Arabia; Industrial University of Ho Chi Minh City, VIET NAM

## Abstract

The evolution of Large Language Models (LLMs) has significantly advanced artificial intelligence, driving innovation across various applications. Their continued development relies on a deep understanding of their capabilities and limitations. This is achieved primarily through rigorous evaluation based on diverse datasets. However, assessing state-of-the-art models in Arabic remains a formidable challenge due to the scarcity of comprehensive benchmarks. The absence of robust evaluation tools hinders the progress and refinement of Arabic LLMs and limits their potential applications and effectiveness in real-world scenarios. In response, we introduce the GATmath (7k questions) and GATLc (9k questions), two Arabic, large-scale, and multitask reasoning and language understanding benchmarks. Derived from the General Aptitude Test (GAT) examination, each dataset covers multiple categories, demanding skills in reasoning, semantic analysis, language comprehension, and mathematical problem-solving. To the best of our knowledge, our dataset is the first comprehensive and large-scale reasoning dataset specifically tailored to the Arabic language. We conducted a comprehensive evaluation and analysis of seven prominent LLMs on our datasets. Remarkably, even the highest-performing model attained a mere 66.9% and 64.3% accuracy, underscoring the considerable challenge posed by our datasets. This outcome illustrates the intricate nature of the tasks within our datasets and highlights the substantial room for improvement in the realm of Arabic language model development.

## Introduction

In recent years, large language models (LLMs) have demonstrated extraordinary abilities in a variety of natural language processing (NLP) tasks [1–4]. Their use has even expanded to address real-world challenges in a variety of fields, such as medicine, finance and education [5–8]. The evaluation of LLMs is a cornerstone in understanding their capabilities, limitations, progress, and areas for improvement [9]. Evaluating LLMs traditionally involves the use of well-structured benchmarks for particular tasks. For example, TruthfulQA [10] measures a model’s truthfulness, whereas FLORES-101 [11] assesses machine translation capabilities. MMLU [12] tests the ability of an LLM to answer a broad range of questions that cover 57 tasks, including elementary mathematics, US history, computer science, and law. These benchmarks provide a reliable framework for evaluating LLM performance on defined tasks. However, as LLMs become increasingly advanced, there is a pressing need for more challenging benchmarks that can comprehensively evaluate the breadth and depth of their abilities while closely mirroring real-world applications and expectations. Therefore, recent efforts have started using tests that were originally designed for humans to evaluate LLM performance. For example, AGIEval [13] was created from general exam questions sourced from the SAT, Gaokao, and GRE exams. Additionally, M3Exam [14] collects official exam questions from end-of-level assessments in primary school, middle school, and high school. Additionally, the MATH dataset [15] consists of problems from mathematics competitions, including AMC 10, AMC 12, and AIME. In fact, human exam questions, which require diverse skills such as critical thinking, reasoning, problem-solving, and integrating knowledge across various domains, offer a rigorous and multifaceted assessment that can be used for improving LLMs [16].

Despite these advancements, most current benchmarks focus predominantly on the English language, which hinders the advancement of language models in other languages. More specifically, the Arabic language stands out as uniquely challenging for computational linguists and researchers in NLP [17]. It poses formidable obstacles due to its rich linguistic features and complex grammatical structure, which is characterised by intricate morphology and syntax.

Arabic presents significant challenges for NLP due to a confluence of linguistic factors [18–21]. These challenges include the following:

1. Morphological Complexity: Arabic’s rich morphology includes gender, number, person, aspect, mood, case, and clitics, resulting in a large number of word forms. This complexity often necessitates multiple English words to translate a single Arabic term, thereby expanding the vocabulary and presenting challenges for machine learning models.
2. Orthographic Ambiguity: The infrequent use of diacritical marks in Arabic leads to ambiguity, as these marks are essential for distinguishing words. While native speakers can often infer meaning from context, the absence of these marks poses challenges for both learners and computational systems. This ambiguity can result in an average of twelve possible interpretations for each word.
3. Dialectal Variation: The use of various dialects in everyday communication adds complexity to NLP in Arabic. While Standard Arabic is used in formal settings, the dialects feature distinct grammars and lexicons. This linguistic diversity makes Standard Arabic NLP tools less effective when applied to dialectal text.
4. Orthographic Inconsistency: This issue, which is common in online communication, affects both Standard and dialectal Arabic. Spelling errors are frequent in online Modern Standard Arabic, and dialectal Arabic lacks a standardised orthography.
5. Resource Limitations: Resource poverty presents a bottleneck. While unannotated text corpora are abundant, resources such as morphological analysers, lexicons, and annotated data are especially limited.

Indeed, accurately assessing the performance of LLMs in Arabic presents a significant challenge due to the limited availability of dedicated datasets for the Arabic language [22]. Currently, evaluating the Arabic proficiency of language models often involves translating English benchmarks into Arabic for assessment [23,24]. However, this approach, while convenient, fails to capture the nuances and intricacies inherent in the Arabic language [25]. Consequently, language models struggle to effectively comprehend and process Arabic text. This shortage of appropriate evaluation resources complicates efforts to enhance LLMs for Arabic, highlighting the need for targeted research endeavours aimed at developing comprehensive, Arabic-specific benchmark datasets that accurately reflect the linguistic and cultural intricacies of this language.

To address these gaps, our work makes three key contributions:

1. We created the GATmath, a large-scale benchmark dataset. It is designed for mathematical reasoning and contains 7,016 Arabic multiple-choice questions (MCQs) spanning four categories: algebra, problem-solving, geometry and comparisons.
2. We also created a second large-scale benchmark dataset named the GATLc. It focuses on language comprehension and reasoning. It has 9,036 Arabic MCQs across five tasks: verbal analogy, sentence completion, contextual error, semantic association, and reading comprehension.
3. We conducted a comprehensive experimental evaluation and analysis using five prominent open-source Arabic-centric models, namely, jais-13b-chat, jais-30b-chat, AceGPT-13B-chat, ALLaM-13B, and ALLaM-70B, and two English LLMs with multilingual abilities: Qwen2-72B and Llama3-70B.

We selected the General Aptitude Test (GAT) dataset as the focal point of our study stems from its specificity to the Arabic language, its comprehensive evaluation of mathematical ability and linguistic proficiency designed for humans, and its quality has been verified by educational experts.

The tested LLMs achieved top performances of 66.9% and 64.3% for the GATmath and GATLc, respectively. This outcome highlights the challenges inherent in our datasets, emphasising the need for LLMs with advanced reasoning skills and a thorough understanding to identify logical connections and draw conclusions across diverse domains.

This paper is organised as follows: Section 2 covers the background. Section 3 discusses related work. Section 4 outlines the process of data collection. Section 5 presents the data statistics. Section 6 describes the evaluation methodology. Section 7 presents the results and discussion. Finally, Section 8 provides the conclusion.

## Background

The GAT exam, which is conducted by the National Center for Assessment [26], is a mandatory standardised test for all prospective college students in Saudi Arabia. Universities set minimum scores for admission, and GAT results significantly impact scholarship awards and selection for specialised programs. Therefore, this test is carefully crafted to be competitive and comprehensive, assessing a wide range of abilities to ensure a holistic evaluation of each candidate’s potential. The GAT exam is divided into two parts: language comprehension and mathematical reasoning. Both sections consist of multiple-choice questions, requiring participants to select the correct answer from four available options. The exam is conducted entirely in Arabic and includes numbers and symbols.

Our methodology of creating two separate datasets is a direct reflection of the bipartite structure of the GAT. Since the exam is designed to independently assess the two different domains, we constructed a unique dataset for each. This approach allows a precise and isolated evaluation of an LLM’s performance in both linguistic understanding and mathematical reasoning, mirroring the established and validated structure of the GAT itself.

### Mathematical reasoning

The mathematical reasoning section of the GAT exam presents a variety of questions that require the application of mathematical methods and concepts. These questions demand a deep understanding of mathematical relationships and the use of appropriate techniques and reasoning to solve problems. Notably, all mathematical symbols, variables, and numbers are presented in Arabic, distinguishing the GATmath benchmark from other datasets. For the GATmath, we classified the questions into four types to identify the strengths and weaknesses of the LLMs and facilitate analysis. These types are:

1. Algebra: This type of question focuses on mathematical operations and algebraic equations, relying heavily on the use of symbols and variables to represent unknown values and solve equations and functions.
2. Problem solving: These questions focus on applying mathematical concepts in practical, real-life scenarios. They include solving problems related to financial issues, ratios, proportions, speed and distance, and time and force.
3. Geometry: Geometry questions focus on the study of shapes, sizes, and properties, including the study of triangles and angles. This section of the aptitude test often features images depicting these shapes. For clarity, in GATmath, we have included only textual geometry questions that do not incorporate images.
4. Comparisons: These questions present two values, and the task is to compare them and choose one of the following options: the first value is greater, the second value is greater, both values are equal, or the information provided is insufficient.

**Fig 1:**
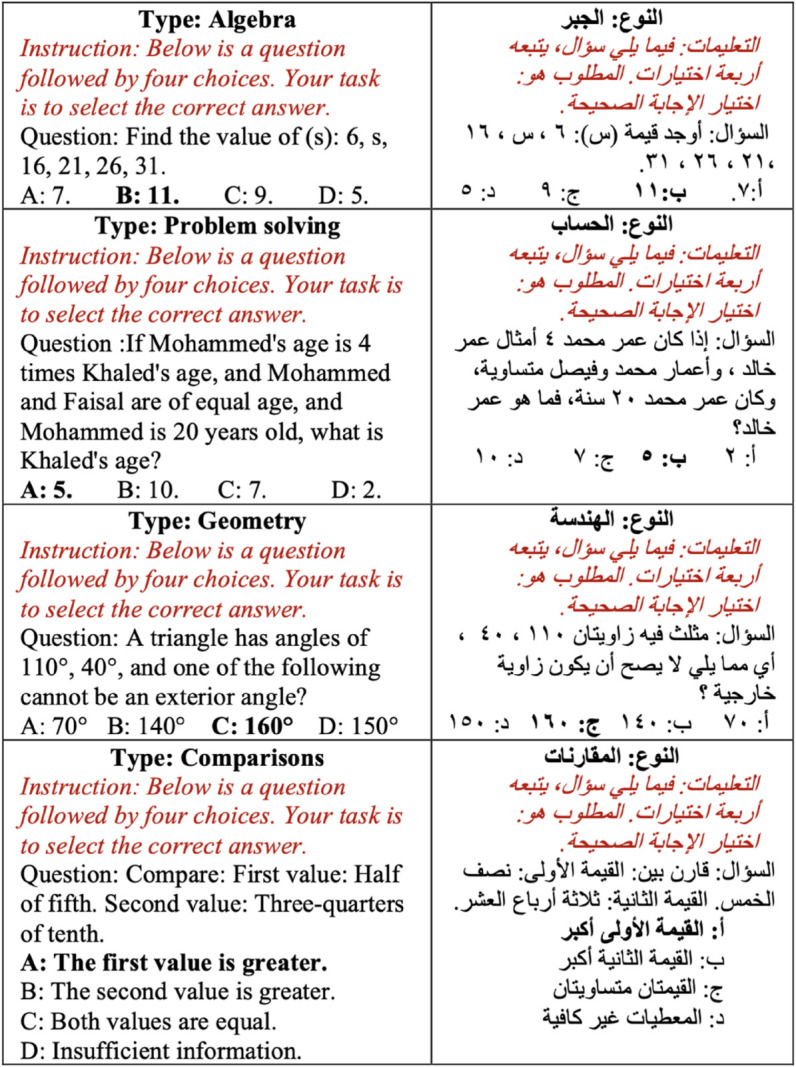
Examples of the four tasks of the GATmath benchmark. The correct choice of each question is indicated in bold. The prompt used is in italics.

Fig 1 shows an example of each type from the GATmath dataset.

### Language comprehension

**Fig 2:**
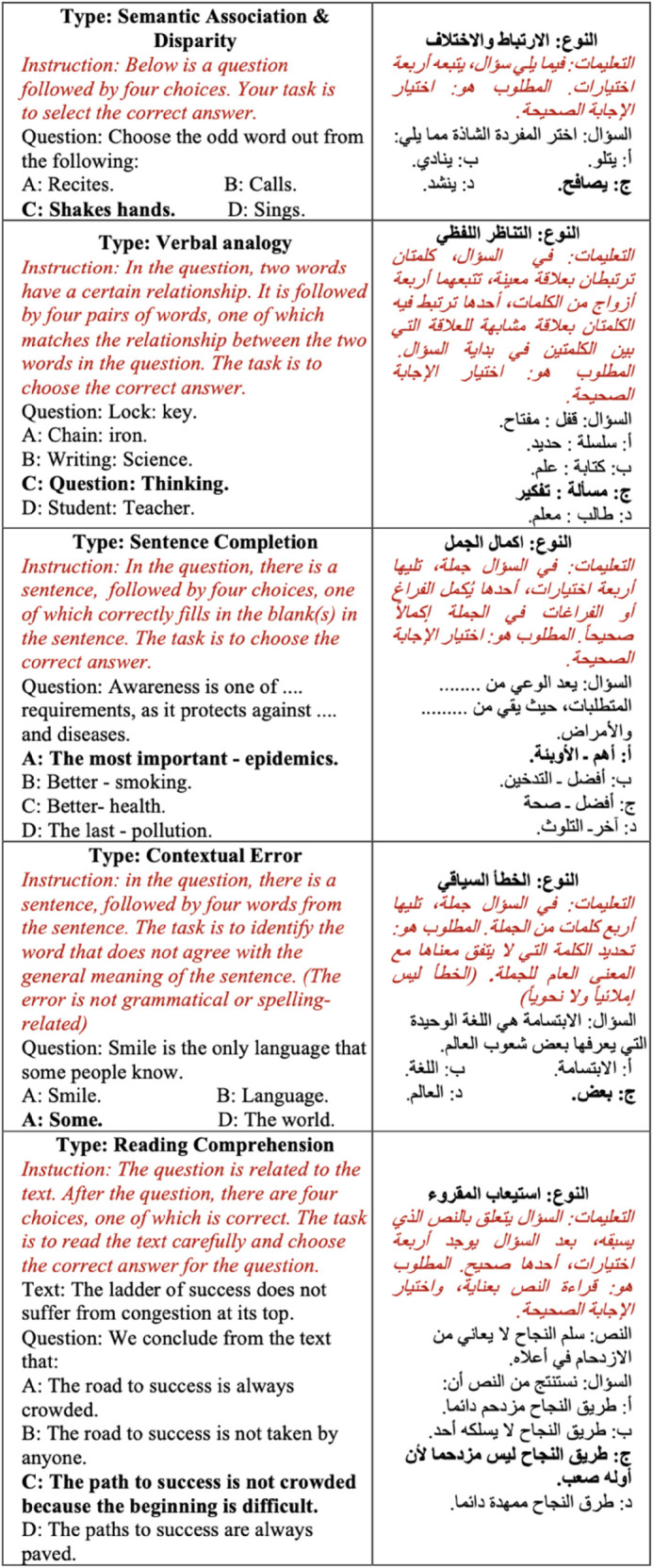
Examples of the five tasks from the GATLc benchmark. The correct choice of each question is indicated in bold. The prompt used is in italics.

This section focuses on understanding and effectively using the Arabic language and assessing abilities across various dimensions. For enhanced analysis, we divide the questions in this section into five tasks. Fig 2 shows an example of each task presented in our GATLc dataset. These tasks are as follows:

1. Verbal analogy: These questions assess the ability to identify relationships between pairs of words. Each question presents a pair of words, and the task is to choose another pair from four options that exhibit the same relationship, such as part-to-whole (e.g., room: house), cause-and-effect (e.g., germs: diseases) and transformation (e.g., water: ice).
2. Sentence Completion: These questions feature sentences with blanks to be filled with appropriate choices from four options, ensuring grammatical accuracy, logical coherence, and consistency in word relationships while enhancing the understanding of cultural proverbs and intellectual awareness.
3. Contextual Error: These questions involve identifying a word inaccurately placed within a sentence, making it unsuitable for the context or contrary to the intended meaning. The task is to identify one word from four options of the text that diverges from or contradicts the overall meaning of the sentence.
4. Semantic Association and Disparity: These questions assess the ability to identify semantic relationships between words or phrases and recognise disparities or anomalies within a given context. The task often involves selecting the word or phrase most closely associated with a given term from four options or identifying the odd term or phrase among a set of words or phrases.
5. Reading Comprehension: This involves understanding and analysing textual material from diverse domains, such as the natural sciences, cultural heritage, and history. It requires responding to questions about various aspects of the text, testing the depth of understanding and critical and analytical thinking capabilities.

## Related work

In recent years, notable advancements have been made in assessing the capabilities of LLMs, largely propelled by the introduction of specialised benchmarks. These resources play a pivotal role in evaluating the scope and proficiency of LLMs across diverse domains. For example, MMLU [12] assesses knowledge understanding through a broad range of four multiple-choice questions covering 57 tasks, such as elementary mathematics, US history, computer science, and law. MMLU-Pro [27] expands the answer choices to ten, incorporates a larger proportion of college-level exam problems, and eliminates inaccurately annotated or unanswerable questions from the MMLU. Additionally, benchmarks such as BIG-bench [28] have extended the assessment scope across 204 different tasks. Some benchmarks have been specifically designed to evaluate particular skills. For example, MATH-V [29] and GSM8K [30] focus on assessing mathematical reasoning at the elementary level. GSM1k [31] was created to mirror the GSM8k benchmark while mitigating data contamination issues. MGSM [32] contains multilingual grade school math. IFEval [33] is tailored for evaluating the ability of LLMs to follow natural language instructions, whereas MUSR [34], HellaSwag [35] and the ARC Challenge benchmark [36] address the assessment of complex reasoning abilities. WinoGrande [37] designed for evaluating commonsense reasoning in natural language understanding. Furthermore, some datasets draw directly from actual human exams, offering a realistic portrayal of diverse scenarios such as M3Exam [14], VNHSGE [16], AGIEval [13] and EXAMS [38].

Recent research in Arabic NLP has placed growing emphasis on the development of native Arabic benchmarks to assess the performance of Arabic LLMs. This focus stems from the recognized limitations of earlier resources, which were often machine-translated from English or other languages. Such translated benchmarks can inadvertently fail to capture the distinct linguistic nuances and rich cultural contexts of the Arabic language [23,39]. Consequently, a number of purpose-built Arabic benchmarks have emerged, each designed to assess specific LLM capabilities within a genuinely Arabic framework. These recent benchmarks evaluate a wide spectrum of abilities. For instance, AraSTEM [40] evaluates LLMs’ knowledge in STEM subjects in Arabic, focusing on technical and scientific comprehension. In a different specialized domain, ArabLegalEval [41] provides a multitask benchmark to assess the understanding and application of complex Arabic legal knowledge. Similarly, MedArabiQ [42] targets the critical area of healthcare, offering a benchmark to evaluate LLMs on Arabic medical tasks.

Beyond domain-specific knowledge, ArabicSense [43] benchmark is designed to test an LLM’s ability to make logical inferences about everyday situations described in Arabic. This is complemented by ArabCulture [44], which delves deeper into culturally-specific commonsense reasoning, assessing a model’s grasp of implicit knowledge embedded within the Arab cultural context. Moreover, several benchmarks address dialectal and cultural diversity. ARADICE [45] and Palm [46] created to evaluate the capabilities of LLMs in understanding and processing various Arabic dialects and their associated cultural expressions. Furthering this effort, Jawaher [47] presents a multidialectal dataset of Arabic proverbs, providing a sophisticated test of both linguistic and cultural fluency. Addressing factual accuracy, the HalluVerse25 [48] benchmark provides a fine-grained, multilingual dataset with a significant Arabic component to detect and analyze model hallucinations.

In terms of existing exam-based Arabic benchmarks that is related to our work, Alkaoud [49] developed a dataset that also sources from the GAT exams, focusing only on the language/verbal components of the exam with only four types, namely, analogy, sentence completion, contextual error, and reading comprehension. In this dataset, the semantic associations and disparity type of questions from the GAT are not presented. This dataset features two versions, one in Arabic and the other in English, comprising 456 and 468 questions, respectively, totalling only 924 questions. The dataset’s design primarily aims to provide a platform to assess the performance of LLMs in processing the same types of verbal questions across both Arabic and English. While this approach offers valuable insights into the different capabilities of models across languages, the dataset’s limited size poses constraints on the scope and thoroughness of analysis possible. Conversely, our datasets incorporate both parts of the GAT exam, in addition to expanding the number of questions to create a robust benchmark that could yield more definitive conclusions regarding the comparative abilities of Arabic LLMs in understanding diverse linguistic inputs.

Reem et al. [50] also introduced ArMATH, an Arabic dataset tailored for solving mathematical word problems. Comprising nearly 6,000 questions, the dataset integrates 3,533 questions that were written using Arabic elementary math books and an additional 2,467 questions translated and localised from the MATH23K dataset [51]. ArMATH focuses on basic arithmetic operations, which are representative of elementary-level mathematics with only a single variable. Even though the word problems are in Arabic, the numbers and variables are presented in English. In contrast, our dataset is larger and enriches the scope by including questions that pertain to high school-level mathematics with multiple variables and concepts, thereby extending the complexity and application of the dataset to more advanced mathematical concepts and problem-solving skills. Moreover, unlike ArMath, all numbers, variables, and symbols in GATmath are represented in Arabic characters. This unique representation underscores the dataset’s specificity, thereby establishing it as an accurate measure tailored specifically for the Arabic language and culture.

ArabicMMLU [22] was recently introduced as a benchmark for knowledge and language understanding in Arabic. It consists of 14,575 multiple-choice questions in Modern Standard Arabic that span areas of history, geography, law, civics education, and driving tests. This dataset mimics the Massive Multi-task Language Understanding (MMLU) [12], which has been widely used as an evaluation benchmark for many new English LLMs. Notably, the math questions in ArabicMMLU represent only 412 elementary-level questions with English numbers, which underscores the uniqueness of our dataset, GATmath, for being entirely in Arabic and larger in size. This comprehensive coverage provides a more rigorous and culturally relevant benchmark for assessing Arabic LLMs.

In the scholarly discourse on multilingual educational datasets, the EXAMs [38] dataset emerged. This dataset encapsulates a variety of school exam questions across multiple languages, including a subset of 562 questions in Arabic. Notably, the Arabic questions in this dataset are direct question-answer types that rely on general knowledge and do not require further skills such as semantic analysis, reasoning, or problem solving.

In this paper, we present the GATmath and GATlc, comprising 7016 and 9036 questions across nine categories. Meticulously designed from human exams, they assess various aspects of the Arabic language alongside mathematical proficiency, thereby providing a robust framework for evaluating cognitive abilities. This unique combination of scale and specificity distinguishes our dataset’s novelty from existing benchmarks and enhances its utility in academic and applied settings.

### Arabic NLP

Recent advancements in Arabic NLP have been driven by a focused effort to overcome the language’s inherent complexities, such as its rich morphology, orthographic ambiguity, and extensive dialectal variation [21]. The most significant recent trend in Arabic NLP is the development of native Arabic LLMs [52], such as Jais [24], ALLaM [53], and AceGPT [23], representing a paradigm shift away from adapting multilingual models. This move is supported by two key data-centric strategies: the creation of massive new Arabic training corpora [54], and the development of sophisticated data augmentation techniques designed to expand smaller, task-specific datasets [55]. These augmentation methods include traditional approaches like back-translation [56] as well as novel, Arabic-specific techniques such as vowel deletion and syntactic style transfer [57]. This dual focus on both creating large-scale resources and maximizing the utility of existing data is fundamental to training the next generation of powerful, culturally-aware Arabic AI.

These advancements in models and data have enabled significant progress in key application areas such as resolving semantic ambiguity and analysis [58–61], Machine Translation [62–64], bias detection [65], code-switching [66], and dialectal Arabic [67].

As these technologies are deployed in high-stakes domains like mental health and finance, a critical discourse on ethics has emerged [68]. Researchers are moving beyond generic AI principles to address issues of cultural misalignment in models trained on Western-centric data, dialectal bias in hate speech detection datasets [69], and the need for responsible, context-aware annotation practices. This growing emphasis on ethical and cultural considerations ensures that the development of Arabic NLP is technically robust, fair, inclusive, and beneficial to the diverse communities of the Arab world.

#### Arabic language models.

Arabic language models can be classified into three main categories: encoder-only, decoder-only, and encoder-decoder models [70]. The encoder-only models, including AraBERT [71], CAMeLBERT [72], AraELECTRA [73], ArBERT, and MARBERT [74], are derived from the BERT [75] family, which learns word context bidirectionally by analysing both preceding and following words. BERT was initially developed for tasks such as text classification and next-sentence prediction. AraT5 [76] is an Arabic encoder-decoder model based on T5 that uses a text-to-text framework and a unique MLM approach for faster training. It also uses adapter modules for task-specific fine-tuning. Decoder-only models such as AraGPT2 [77] operate in a unidirectional manner, predicting sequential outputs by focusing on the preceding context. This makes them ideal for text creation and language processing [78]. Other notable decoder-only models include Jais and Jais-chat [24], which were trained on a massive Arabic corpus of 73 billion tokens and fine-tuned for instruction-following tasks. AceGPT [23], which was further enhanced through reinforcement learning from human feedback in Arabic. The LaMA3-70b-instruct model [79], a 70 billion-parameter model from Meta AI’s LLaMA3 series, is optimised for complex instruction following. Moreover, the ALLaM model [53] was derived from LLaMA, which aims to advance Arabic Language Technologies (ALT) through sophisticated training techniques focused on language alignment and knowledge transfer, including cultural values relevant to the Arabic-speaking world. Furthermore, Arabic is included in multilingual models that are trained on Arabic data. This includes Aya [80], an instruction-finetuned model built on the mT5 architecture. BLOOM [81] is a model with 176 billion parameters trained across 46 languages. Additionally, the Qwen2 model [82] is instruction-tuned trained on Arabic data designed to excel in understanding and following instructions with different size variants from 0.5B to 72B parameters.

## Dataset collection

The compilation of the datasets was challenging, as it relied on image-based PDFs from publicly available GAT preparation platforms. The inadequacy of Arabic language support in existing conversion tools necessitated manual transcription of the PDFs. The detailed organisation of these PDFs involved careful selection, categorisation by year and type, and detailed numbering to reduce duplication and facilitate future processing, thereby streamlining collaboration by freelancers and periodic review from the authors.

**Fig 3:**
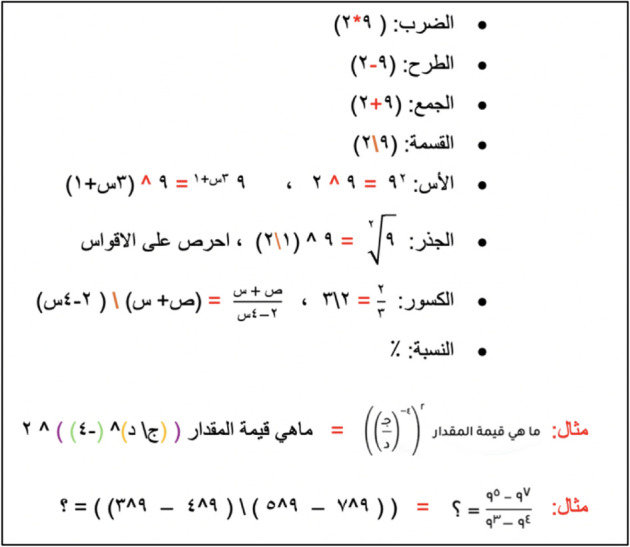
Transcription guide for GAT mathematical questions.

A comprehensive guide was created to standardise the conversion process and ensure consistent quality across contributions. To accommodate the specific challenges of transcribing math questions into textual formats, we provided detailed explanations of all mathematical operations, accompanied by examples to clarify any potential ambiguities. Fig 3 shows examples from the created guide.

**Fig 4:**
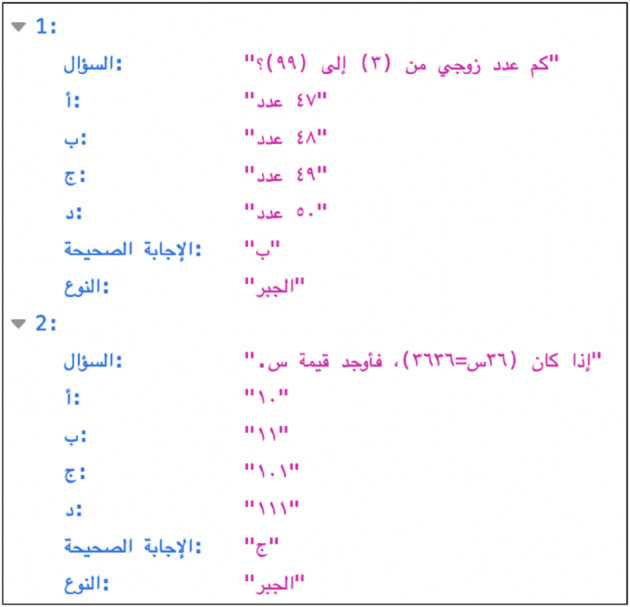
Two objects of the created JSON file.

We engaged with over 90 freelancers from Upwork (https://www.Upwork.com) and Bahr (https://bahr.sa/). We provided them with a detailed guide and requested the completion of a sample set of 5 questions. This step ensured that they understood project requirements, followed the guidelines precisely, and effectively managed mathematical notation. Following a thorough review of submissions, we selected 5 freelancers for the GATmath dataset collection and another 5 for the GATLc. Each freelancer was assigned specific files, numbered accordingly, to work on. The workflow was designed to handle submissions in batches of 300 questions to allow for effective oversight and quality control, ensuring that each batch adhered to the project’s standards before proceeding to the next batch. This methodical approach allowed us to closely monitor progress, provide timely feedback, manage the workflow efficiently, and uphold the integrity and accuracy of the transcribed dataset. Subsequently, the transcribed data were formatted into JSON (Fig 4) with an emphasis on human readability and interaction with the data with proper indentation and spacing, in contrast with other datasets where JSON objects are compacted into a single line.

For the revision phase, we selected two expert freelancers: one with a strong mathematical background to oversee the GATmath questions and another with linguistic expertise to review the GATLc. Assigning only one freelancer per dataset was aimed to ensure consistency within each area. The revision process involved comparing the original PDFs with the transcribed questions and thoroughly checking for mathematical notation accuracy and linguistic precision. Freelancers submitted revisions in segments of 300 questions, including a report of changes, to maintain a structured workflow. After receiving our feedback and approval, they proceeded to the next set. This thorough revision was crucial to ensure the accuracy, consistency, and overall integrity of the dataset, upholding the high standards expected for the GAT exam. Next, the revised batches were amalgamated for each section, yielding a cumulative count of 7,754 math questions and 10,281 language comprehension questions. After that, we conducted an automated filtration process to eliminate duplicates. This procedure resulted in reducing the initial pool for the GATLc and GATmath to 9036 and 7016 questions, respectively, effectively representing the comprehensive scope of the GAT exam.

## Data statistics

Both datasets were categorised into separate files based on the previously delineated tasks. This systematic organisation aims to streamline future retrieval, analysis, and application within educational and research domains. Additionally, this categorisation process facilitates a more comprehensive evaluation of LLMs, allowing for a nuanced assessment of areas necessitating improvement and enabling more thorough analysis. Tables 1 and 2 present the distributions of the GATmath and GATLc across four and five tasks for each dataset, respectively.

**Table 1:** GATmath distribution across four tasks.

Type	Number of questions
Algebra	2700
Problem-solving	2721
Geometry	370
Comparisons	1225
**Total**	**7016**

**Table 2:** GATLc distribution across five tasks.

Task	Number of questions
Verbal analogy	2812
Sentence Completion	1215
Contextual Error	1309
Semantic Assoc.	1050
Reading Comp.	2650
**Total**	**9036**

## LLMs evaluation

We evaluated the performance of seven prominent Arabic language models using our newly curated Arabic benchmarks, GATmath and GATLc. The models assessed are jais-13b-chat, jais-30b-chat [24], AceGPT-13B-chat [23], LLaMA3-70b-instruct model [79], ALLaM-13B, ALLaM-SFT-70B [53], and Qwen2-72B-Instruct [82]. The evaluation metric that we use is accuracy, as shown in the following equation:


$$\text{Accuracy of task A}=\frac{\text{Number of Correct Answers by the LLM in task A}}{\text{Total Number of Questions in task A}}\times 100$$

**Fig 5:**
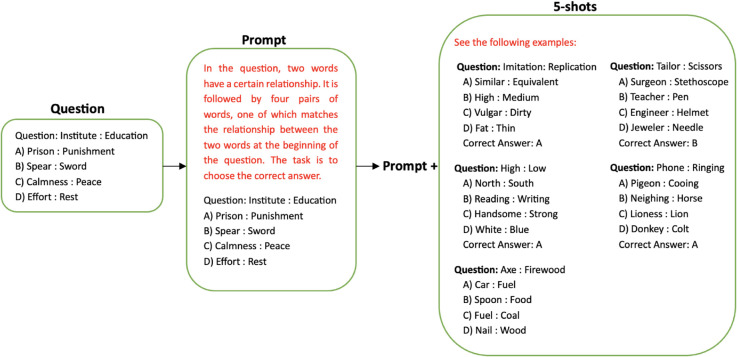
An example of the five-shot evaluation settings used.

In our evaluation, we used a 5-shot in-context learning (ICL) paradigm to assess the model’s ability to adapt to new tasks from a limited number of examples. This approach is predicated on providing the model with five illustrative input-output pairs (the support set) within the prompt itself, followed by the actual query for which a prediction is sought. The model is then expected to generalize from these examples to generate the correct output for the unseen query. Fig 5 shows an example of the evaluation settings used, with the original Arabic prompts translated into English for better clarity.

The architecture of our 5-shot learning implementation is intrinsically tied to the underlying pre-trained language model utilized in our experiments. In this paradigm, the model itself functions as the learning architecture, and no task-specific fine-tuning of the model’s weights is performed. The architecture can be conceptualized as the comprehensive structure of the input prompt that is processed by the model in a single forward pass.The input to the model is a structured prompt, which consists of two primary components:

1. **The Support Set (𝒮)**: This set is composed of five distinct examples, where each example (*s*_*i*_) is an input-output pair:
  $$𝒮=\{s_{1},s_{2},s_{3},s_{4},s_{5}\},\;\text{with }s_{i}=(x_{i},y_{i}).$$
  Here, *x*_*i*_ represents the input prompt for the *i*-th example, and *y*_*i*_ is its corresponding ground-truth output. These examples are curated to be representative of the task the model is expected to perform.
2. **The Query (𝒬)**: Following the support set, the prompt includes the new, unseen input, or query (*x*_*q*_), for which the model must generate a prediction.

The complete prompt (*P*) fed to the model is the concatenation of the support set and the query, typically formatted as follows:


$$P=[s_{1},s_{2},s_{3},s_{4},s_{5},x_{q}]$$

The training in this context is an inference-time process where the model learns the task’s underlying patterns directly from the support set. The features it learns are not pre-defined but are instead semantic and syntactic regularities, such as the relationship between inputs and outputs and the expected response format. The model’s attention mechanism is then able to apply these inferred patterns from the examples to the query to generate the final prediction.

We also used the lm-evaluation-harness [83] to conduct our specific tasks. lm-evaluation-harness is a standardised framework designed for assessing the performance of language models across a wide range of tasks. This framework allows for a consistent and scalable evaluation, providing mechanisms to rigorously test language models against predefined benchmarks and datasets. It is particularly effective for comparative analysis across different models, as it ensures that each model is evaluated under the same conditions and with the same input data. By incorporating our dataset as a new task within the lm-evaluation-harness, we facilitate the evaluation and improvement of LLMs. This approach enhances the accessibility and usability of datasets in the broader research community, enabling further exploration and development of Arabic LLMs.

## Results and discussion

We carefully standardised the prompts for each category of questions in both datasets to replicate those used in the official GAT examination [26]. By adhering to the original prompts, we aim to avoid the risk of adding biases and refrain from engaging in manipulative practices related to prompt formulation. These prompts, which are tailored to each question category, are depicted in Figs 1 and 2, presented in italicised format preceding the respective questions. The results of the GATmath evaluation are illustrated in Table 3 for each of the four tasks, while Table 4 displays the evaluation results for the GATLc across the five tasks. Note that we conducted the experiments using a five-shot setting, wherein the model is provided with five examples from each task to learn from before making predictions. This approach contrasts with zero-shot learning, where the model must make predictions without any prior examples. The five-shot method allows the model to grasp some context and patterns from the provided examples, potentially leading to better performance than in zero-shot scenarios. However, the observed accuracy remained low, underscoring the complexity of the datasets. This finding supports the assertion that our datasets present a significant challenge for LLMs, thereby serving as a valuable resource for testing and enhancing the capabilities of language models.

**Table 3:** Performance of Arabic LLMs on various tasks of GATmath dataset.

Model	Algebra	Problem-solving	Geometry	Comparisons	Avg
jais-13b-chat	0.253	0.266	0.268	0.259	0.262
jais-30b-chat	0.298	0.276	0.308	0.358	0.310
AceGPT-13B-chat	0.262	0.240	0.278	0.266	0.262
Llama3-70b-instruct	0.480	0.455	0.500	0.438	0.468
ALLaM_13B	0.442	0.402	0.435	0.389	0.417
ALLaM-SFT-70B	0.601	0.617	0.581	0.499	0.574
Qwen2-72B-Instruct	0.694	0.691	0.705	0.585	0.669

**Table 4:** Performance of Arabic LLMs on various tasks of the GATLc dataset.

Model	Analogy	Sentence Comp.	Con. Error	Association	Reading Comp.	Avg
jais-13b-chat	0.326	0.393	0.233	0.231	0.431	0.323
jais-30b-chat	0.331	0.535	0.273	0.432	0.489	0.412
AceGPT-13B-chat	0.323	0.409	0.263	0.343	0.401	0.348
Llama3-70b-instruct	0.391	0.618	0.365	0.574	0.547	0.499
ALLaM_13B	0.435	0.657	0.451	0.528	0.572	0.528
ALLaM-SFT-70B	0.529	0.777	0.515	0.669	0.646	0.627
Qwen2-72B-Instruct	0.590	0.774	0.585	0.640	0.624	0.643

### GATmath analysis

As illustrated in Table 3, the evaluation results demonstrate a clear correlation between model size and overall performance. Compared with their smaller counterparts, larger models, such as Qwen2-72B-Instruct, generally achieve superior results. Nevertheless, these advanced models still face significant challenges when dealing with the complexity of the new dataset. Next, we provide a detailed analysis of each model’s performance.

- **Qwen2-72B-Instruct**: This model exhibits the best performance among all the models, with an average score of 66.9%. This model’s relatively high performance underscores its robust capabilities across different mathematical tasks. Its consistent performance across most of the categories suggests that it has a well-rounded understanding and ability to address diverse mathematical problems. However, for the ’Comparisons’ task, its performance is comparatively low at 58.5%. This is an indicator that the model, though good at procedural calculation (Algebra, Geometry), is poor when the task is more about abstract relational reasoning and determining the adequacy of information offered, a higher-level form of reasoning. The fact that it still does not exceed 66.9% on average indicates the challenging nature of the dataset, emphasising its potential to push the boundaries of existing models.
- **ALLaM-70B**: The ALLaM-70B model achieves an average score of 57.4%. This model also demonstrates balanced performance across 3 tasks. Nevertheless, its lower performance in comparisons indicates a potential gap in understanding comparative relationships and contextual evaluations. This discrepancy highlights the intricate nature of comparison tasks within the dataset, making it a critical area for future model improvements.
- **Llama3-70b-instruct**: This model performs moderately well, with an average score of 46.8%. Its strengths lie in geometry and algebra, reflecting a better grasp of spatial and structural reasoning. However, its comparatively lower performance in comparisons and arithmetic suggests difficulties in maintaining accuracy and contextual understanding across these domains.
- **ALLaM-13B**: ALLaM-13B has an average performance of 41.7%, reflecting a balanced yet moderate capability across all tasks. Its uniform scores suggest that while it does not excel in any particular area, it also does not significantly underperform in any specific category. This consistency indicates stable but limited proficiency in handling the diverse mathematical challenges presented by the dataset. The model’s performance can serve as a baseline for evaluating improvements in future iterations, particularly focusing on enhancing its capabilities to achieve higher accuracy and understanding.
- **jais-30b-chat**: This model achieves an average score of 31%, indicating significant difficulty with the dataset. Interestingly, it achieves the highest score in comparisons among all tasks, despite the above models. These findings suggest that jais-30b-chat has strengths in understanding and evaluating comparative relationships. However, its performance in other areas, such as arithmetic and algebra, remains very weak, highlighting the need for substantial advancements in model training and architecture to improve overall performance.
- **jais-13b-chat and AceGPT-13B-chat**: Both jais-13b-chat and AceGPT-13B-chat exhibit the lowest performance, each with an average score of 26.2%. Their uniform performance across categories indicates a general struggle with the dataset’s complexity. The low scores of these models reinforce the dataset’s role as a rigorous benchmark.

### GATLc analysis

The accuracy results, once again, reveal a correlation between model size and overall performance. Larger models, with 70 billion parameters or more, tend to outperform smaller models. However, these advanced models still face significant challenges when dealing with the complexity of the new dataset, which achieves the highest average accuracy of only 62.7%.

- **Qwen2-72B-Instruct**: As noted in GATmath, this model exhibits the highest performance among all the models, with an average score of 64.3%. Its performance varies across the five tasks, indicating different strengths and weaknesses. The model excels in sentence completion, with a score of 77.4%, showcasing its proficiency in grammatical accuracy and logical coherence. However, its performance in analogy (59.0%) and contextual error (58.5%) is relatively lower, suggesting difficulty in identifying relationships and contextually inappropriate words. Despite its overall robust capabilities, the model’s average score below 64.3% highlights the challenging nature of the dataset and areas for potential improvement even in advanced models.
- **ALLaM-70B**: Close behind is the ALLaM-70B model, which achieves an average score of 62.7%. This model has particular strengths in sentence completion, reading comprehension, and association, indicating effective training in tasks requiring grammatical accuracy, comprehensive textual analysis, and semantic connections. Notably, it outperforms Qwen2-72B-Instruct in these three tasks. However, it performs poorly on ’Contextual Error’ and ’Verbal Analogy’. This might be because, while the training of ALLaM aimed at Arabic knowledge and culture, it might not have dealt effectively with the kind of abstract, second-order relation mapping involved in analogy, somewhat beyond literal meaning.
- **Llama3-70b-instruct**: The Llama3-70b-instruct model achieves a moderate average score of 49.9%, with varied performance across the five tasks. It shows considerable strength in sentence completion, with a score of 61.8%, reflecting its proficiency in ensuring grammatical accuracy and logical coherence. However, its performance in contextual error (36.5%) and analogy (39.1%) is comparatively lower, indicating difficulties in identifying relationships between pairs of words and pinpointing contextual errors.
- **ALLaM-13B**: The ALLaM-13B model achieves an average score of 52.8%, exhibiting a performance pattern similar to its larger counterpart, ALLaM-70B, but has lower accuracy across all tasks.
- **jais-30b-chat**: The jais-30b-chat model achieves an average score of 41.2%, indicating significant difficulty with the dataset. Its slightly better performance in sentence completion suggests some understanding of the sentence structure, but overall, the model struggles with achieving high accuracy. Notably, it performs worst in contextual error, with a score of 27.3%, highlighting severe difficulties in identifying words that do not fit the context.
- **jais-13b-chat and AceGPT-13B-chat**: Both jais-13b-chat and AceGPT-13B-chat exhibit lower performance, with average scores of 32.3% and 34.8%, respectively. Their uniform performance across categories indicates a general struggle with the dataset’s complexity. These models face considerable challenges, particularly with respect to contextual error. The low scores of these models reinforce the low scores achieved by these models, highlighting the dataset’s demanding nature and underscoring the necessity for advanced methods to address its complexities effectively.

**Fig 6:**
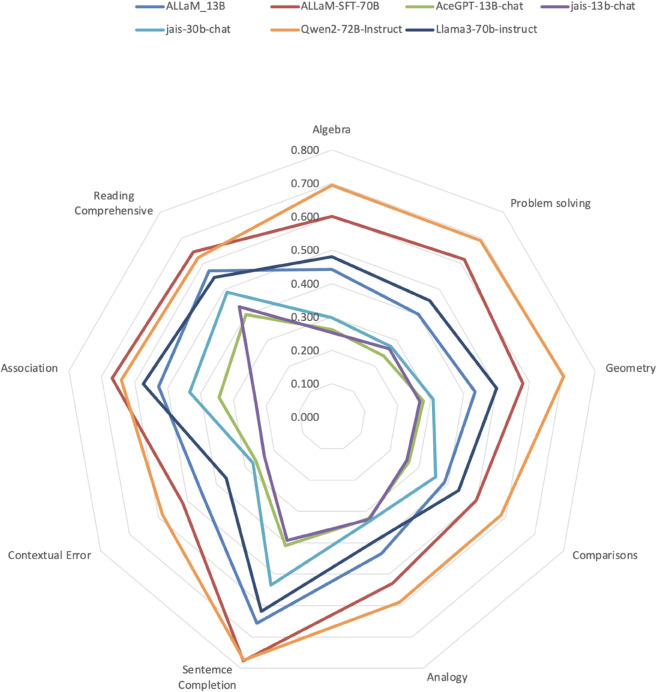
Accuracy of the Models on the Nine Tasks of GATLc and GATmath Datasets.

Fig 6 visualises the accuracy of the models across all nine tasks of the GATLc and GATmath datasets.

To provide broader insight, Table 5 presents the results of some of the tested models on other recent Arabic benchmarks.

**Table 5:** Performance of models on other Arabic benchmarks. CS: Commonsense.

Dataset	Tested Ability	jais 13b chat	jais 30b chat	Qwen 72b Inst	Llama 3	Llama3 8B Inst	Llama3 70B Inst	Ace gpt 13B Chat
AraSTEM [40]	STEM Knowledge	–	58%	–	–	54%	–	46%
ArabicSense [43]	CS	–	–	–	77.5%	–	–	–
ArabLegalEval [41]	Legal Knowledge	–	–	71.47%	–	–	–	–
ArabCulture [44]	CS	54.4%	54%	80%	–	–	34.3%	–
ARADICE [45]	Cultural	–	–	–	–	–	–	–
HalluVerse25 [48]	Halluc.	–	–	65.22%	–	–	65.7%	–
MedArabiQ [84]	Medical	–	–	40.1%	34.3%	–	–	–
ArabicMMLU [22]	Knowledge	54.8%	62.3%	–	–	–	–	52.6%
GATmath	Math	26.2%	31.0%	66.9%	–	–	46.8%	26.2%
GATLc	Language reasoning	32.3%	41.2%	64.3%	–	–	49.9%	34.8%

### Comparative analyses with other languages

Notably, while some LLMs demonstrate strong performance on established English-language benchmarks for mathematical reasoning abilities, their performance on the Arabic math benchmark developed for this study reveals significant disparities. As illustrated in Table 6, the Llama3-70b-instruct model [85,86], which achieved 93% accuracy on the GSM8K and 86.9% on MGSM English math benchmarks, exhibited substantially lower performance on the GATmath (46.8%). Similarly, Qwen2-72B-Instruct model [82] achieving 91.1% on GSM8K and 82.40% on MGSM, its performance on the GATmath was considerably lower, at 66.9%.

**Table 6:** Performance of models on mathematical reasoning benchmarks.

Model	GSM8K	MGSM	GATMath
Llama3-70b-instruct	93%	86.9%	46.8%
Qwen2-72B-Instruct	91.1%	82.4%	66.9%

This pattern is seen again in the GATLc benchmark, where all models exhibit a significant drop in performance compared to their scores on English-language benchmarks (Table 7). For instance, Llama3-70B-instruct [86] achieved 87.5% on FEval, 86% on MMLU, 94.8% on ARC Challenge, 85.5% on HellaSwag and 83.3% on WinoGrande, yet only scored 49.9% on GATLc.

**Table 7:** Performance of models on reasoning and language comprehension benchmarks.

Model	FEval	MMLU	ARC Challenge	HellaSwag	WinoGrande	GATLc
jais-13b-chat	–	–	46.8%	77.6%	–	32.3%
jais-30b-chat	–	–	51.1%	78.9%	–	41.2%
AceGPT-13B-chat	47.33%	63.99%	49.2%	–	–	34.8%
Llama3-70b-instruct	87.5%	86%	94.8%	85.5%	83.3%	49.9%
Qwen2-72B-Instruct	91.1%	82.3%	68.9%	87.6%	82.79%	64.3%

A similar trend appears in Qwen2-72B-Instruct [82], which attained 91.1% on FEval, 82.3% on MMLU, and 87.6% on HellaSwag, but only 64.3% on GATLc.

Even AceGPT-13B-chat [23], which performed moderately on FEval (47.33%) and MMLU (63.99%), dropped to 34.8% on GATLc. Meanwhile, the Jais models, which are tailored for Arabic, showed relatively low performance across the board: Jais-13B-chat and Jais-30B-chat [24] scored 46.8% and 51.1% on ARC Challenge, but only 32.3% and 41.2% on GATLc, respectively.

**Fig 7:**
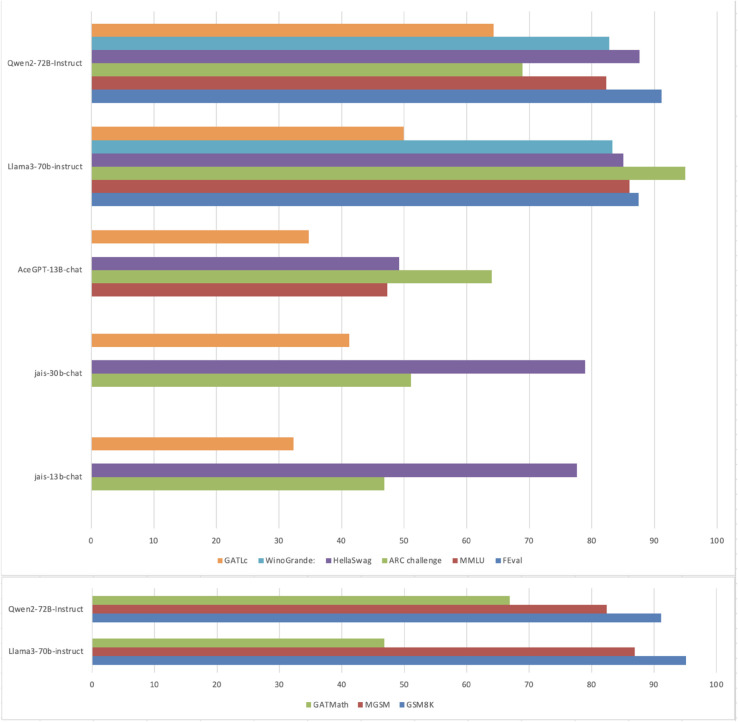
Performance of LLMs on GATmath and GATLc compared with English benchmarks.

These results reinforce the observed gap in cross-lingual generalization, revealing that strong reasoning abilities in English do not necessarily translate to high performance on complex Arabic language tasks. Fig 7 visualizes the above comparison.

### Comparative discussion of GATmath and GATLc results

The differing ranks of the models in GATmath and GATLc highlight the varying strengths and weaknesses of each model when confronted with mathematical reasoning versus linguistic comprehension tasks. This comparative discussion aims to bridge the two sections, emphasising the interplay between model capabilities and dataset complexity.

#### Performance trends across datasets.

A consistent trend across both datasets is the correlation between model size and performance. Larger models, such as Qwen2-72B-Instruct and ALLaM-70B, consistently outperform smaller models in both the GATmath and GATLc. However, the degree of superiority varies significantly between the two datasets. For example, Qwen2-72B-Instruct achieves an average score of 66.9% on the GATmath but decreases to 64.3% on the GATLc, suggesting that while it performs relatively better in mathematical reasoning, it still faces significant challenges in linguistic tasks. Similarly, ALLaM-70B performs better in GATLc (62.7%) than in GATmath (57.4%), indicating that its strengths may be more aligned with linguistic analysis than with mathematical problem solving. This contrast highlights the varying demands of the two datasets and the specialised capabilities required for each.

#### Model performance across domains.

A notable difference in model rankings can be observed between the two datasets. For example, Llama3-70b-instruct achieves a higher average score than ALLaM-13B on the GATmath (46.8% vs. 41.7%). However, with the GATLc, this trend reverses, with ALLaM-13B scoring higher (52.8%) than Llama3-70b-instruct (49.9%). These differences indicate that models may have domain-specific strengths, even as their overall accuracy remains low.

#### Task-specific challenges.

The comparative analysis also highlights task-specific challenges that models face across the two datasets. In the GATmath, tasks requiring comparative analysis and decision-making (e.g., comparisons) prove particularly challenging for all the models, with even the top-performing Qwen2-72B-Instruct achieving only 58.5%. In contrast, with the GATLc, tasks such as contextual error and analogy emerge as the most difficult, with even the best models struggling to achieve scores above 60%. This suggests that while mathematical reasoning demands precise logical and structural understanding, linguistic tasks require a deeper grasp of contextual relationships and semantic nuances, which remain challenging for current models.

## Conclusion

In this study, we introduced the GATmath and GATLc, two novel, large-scale Arabic benchmarks designed to evaluate the mathematical reasoning and Arabic language understanding capabilities of LLMs. Derived from the GAT exam, these datasets encompass a diverse range of tasks requiring complex cognitive skills. Our comprehensive evaluation of seven prominent LLMs revealed a significant gap in their ability to handle these challenging questions, with the top performance reaching only 66.9% and 64.3% on the GATmath and GATLc, respectively. These findings underscore the need for further advancements in LLM capabilities to address the complexities of the Arabic language and reasoning tasks. We believe that GATmath and GATLc offer valuable resources for the research community to develop more robust and capable language models, ultimately contributing to the progress of Arabic NLP. For future work, we plan to expand the datasets to include image-based questions from the GAT exams, such as charts and geometrical figures, and then test the performance of vision-LLMs. Moreover, conducting a comprehensive error analysis to identify and understand the specific error types and patterns remains an area for future exploration.
