# Correction: GATmath and GATLc: Comprehensive benchmarks for evaluating Arabic large language models

**DOI:** 10.1371/journal.pone.0338465

**Published:** 2025-12-09

**Authors:** The PLOS One Staff

## Notice of Republication

This article was republished on November 14, 2025, to correct errors in the Data Availability Statement. The publisher apologizes for the errors. Please download this article again to view the correct version. The originally published, uncorrected article and the republished, corrected articles are provided here for reference.
